# 

**Published:** 1842-12-03

**Authors:** 


**CONTENTS OF No. 49.**

Dr. Q. Gibbon's Case of Idiopathic Tetanus, cured by Spirits of Turpentine, - - - - - - - - - - - 769
Dr. John M. Turner's Anomalous cases of Pregnancy, with Remarks, 770

BIBLIOGRAPHICAL NOTICE: On certain Medical Delusions. An Introductory Lecture to the Course of Institutes of Medicine in Jefferson Medical College of Philadelphia. Delivered November 4, 1842. By Robley Dunglison, M. D., - - - - 773

ANALECTA: Fracture of the Spine and Sternum, - - - - - 775
Cure of Sore Nipples, - - - - - - - - 776
Post Mortem Examination of the Genito-Urinary Organs, 777
Diseases that never co-exist, - - - - - - 777
Dr. Watson on Cancer of the Stomach, - - - - 778
Dr. Stricker on the Hospitals of Italy, - - - - - 782

